# Bumblebees Learn a Relational Rule but Switch to a Win-Stay/Lose-Switch Heuristic After Extensive Training

**DOI:** 10.3389/fnbeh.2020.00137

**Published:** 2020-08-12

**Authors:** HaDi MaBouDi, Cwyn Solvi, Lars Chittka

**Affiliations:** ^1^School of Biological and Chemical Sciences, Queen Mary University of London, London, United Kingdom; ^2^Department of Computer Science, University of Sheffield, Sheffield, United Kingdom; ^3^Department of Biological Sciences, Macquarie University, North Ryde, NSW, Australia

**Keywords:** abstract concepts, adaptive decision-making, animal cognition, behavioral analyses, cognitive flexibility, cognitive offloading, the law of least effort

## Abstract

Mapping animal performance in a behavioral task to underlying cognitive mechanisms and strategies is rarely straightforward, since a task may be solvable in more than one manner. Here, we show that bumblebees perform well on a concept-based visual discrimination task but spontaneously switch from a concept-based solution to a simpler heuristic with extended training, all while continually increasing performance. Bumblebees were trained in an arena to find rewards on displays with shapes of different sizes where they could not use low-level visual cues. One group of bees was rewarded at displays with larger shapes and another group at displays with smaller shapes. Analysis of total choices shows bees increased their performance over 30 bouts to above chance. However, analyses of first and sequential choices suggest that after approximately 20 bouts, bumblebees changed to a win-stay/lose-switch strategy. Comparing bees’ behavior to a probabilistic model based on a win-stay/lose-switch strategy further supports the idea that bees changed strategies with extensive training. Analyses of unrewarded tests indicate that bumblebees learned and retained the concept of relative size even after they had already switched to a win-stay, lost-shift strategy. We propose that the reason for this strategy switching may be due to cognitive flexibility and efficiency.

## Introduction

Cognitive flexibility reflects an individual’s ability to adaptively alter their behavioral strategy following a changing environment (Wasserman and Zentall, 2006). A fundamental challenge for animal cognition researchers is to decipher which strategies an animal uses in solving any particular task (Shettleworth, 2001; Chittka et al., 2012). Indeed, there are often multiple ways for an animal to solve a behavioral task.

Bees have been shown capable of learning various abstract relationships, for example rules about target size (e.g., “pick the larger (or smaller) of two object sizes”), amongst myriad impressive cognitive abilities (Perry and Barron, 2013; Chittka, 2017; Skorupski et al., 2018). However, in some of these cases, it may be that bees use a variety of different strategies to solve the tasks they are confronted with Cope et al. (2018) and Skorupski et al. (2018). One recent study showed that bees can solve a spatial concept learning task using a simple visual discrimination strategy through sequential scanning of stimuli rather than needing to compare stimuli based on an abstract rule, though some individuals may well follow such a rule (Guiraud et al., 2018). In numerical cognition tasks, honeybees may also use alternative cues that correlate with a number, but are not in themselves numerical (Vasas and Chittka, 2019; MaBouDi et al., 2020). Bees’ behavior in solving a delayed matching-to-sample task is replicated by a model without any neural representations of the abstract concepts of sameness or difference (Cope et al., 2018). Even the same individuals may have recourse to different solutions to the same task, depending on the extent of training. For example, with an increased number of training trials with a single pair of patterns, individual honeybees have been shown to have a greater generalized response to novel stimuli, i.e., the representation necessary to discriminate subsequent visual patterns changes with extended training (Stach and Giurfa, 2005). All of these findings highlight the need for considering alternative strategies used by animals in cognitive tasks. This does not just concern the traditional dichotomy of “simple” vs. “complex” solutions to such tasks. Different individuals may use different solutions that are equal in complexity, depending on their particular path to figuring out a solution.

Previous works have shown that honeybees can solve a task that appears to necessitate learning the concept of relative size and apply the rule to novel sizes within or outside the size range they were trained (Avarguès-Weber et al., 2014; Howard et al., 2017). As with the examples above, bees may use more than one strategy to solve the same task, depending on the training protocol and context. Here, we test bumblebees to determine the strategies by which they cope with a relational rule learning task (“larger-than”/“smaller-than”) and examine their behavior over time to reveal the cognitive strategies used throughout the training.

## Materials and Methods

### Animals and Experimental Setup

Bumblebees (*Bombus terrestris audax*) from commercially available colonies (Agralan Limited, UK), were housed in a wooden nest-box connected to a flight arena (100 cm × 75 cm × 30 cm). Bees were allowed access to a flight arena through an acrylic corridor (25 cm × 3.5 cm × 3.5 cm). Three plastic sliding doors located along the corridor allowed controlled access to the arena. The arena was covered with a UV-transparent clear acrylic sheet. The stimuli were presented to bees on the gray-colored back wall of the arena. Colonies were provided with ~7 g irradiated commercial pollen (Koppert B.V., The Netherlands) every 2 days. Bees from three colonies were used in this study.

### Pretraining Phase

**Figure 1:**
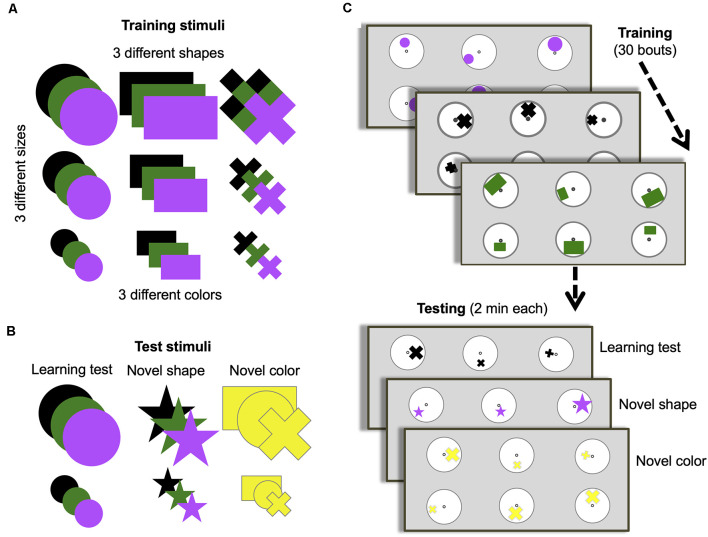
Training and testing protocol. **(A)** Stimuli options used during training. **(B)** Stimuli options used for each of the three different unrewarded tests. **(C)** Training and test protocol. Bees were trained for 30 bouts (visits to the arena before returning to the hive). All stimuli in panel **(A)** were used randomly across bouts during training. Only two of the possible three sizes of shapes were presented during a single bout. Only one of the possible three colors and one of the possible three shapes were presented each bout. One group of bees (*n* = 10) was trained to find a 50% sucrose solution at the center of the stimulus containing the larger of the three shapes and bitter quinine solution at the smaller of the three shapes. Another group (*n* = 8) were trained on the opposite contingency. Once training was complete, bees were subjected to three unrewarded tests (with one or two reminder/training bouts between each test to keep bees motivated). All tests used small and large-sized shapes. The learning test used one randomly chosen type and color used during training. The novel shape test used one randomly chosen color used during training but always a star shape that had not been used during training. The novel color test used one randomly chosen shape used during training but always colored yellow, which had not been used during training.

All bumblebee workers were recruited from a gravity feeder containing 30% (w/w) sucrose solution placed in the center of the arena. Outside of experiments, the colony was provided with a 30% (w/w) sucrose solution from a small gravity feeder placed inside the nest-box during the evenings. Successful foragers on the arena gravity feeder were individually marked with number tags, superglued to their thorax, for identification during the subsequent experiment (Opalithplättchen, Warnholz and Bienenvoigt, Ellerau, Germany). Each day of experimentation, marked bees were pre-trained to find 50% (w/w) sucrose solution from microcentrifuge tubes (5 mm diameter) at the center of each of six white discs (7 cm diameter) on the gray-colored back wall of the arena, horizontally 14 cm from each other vertically 9.3 cm (positioned as in Figure 1). These discs were made of paper and covered with a transparent laminate to enable cleaning with 70% ethanol in water (v/v). All stimuli were printed with a high-resolution printer.

### Training Phase

Each day, after several number-tagged bees had learned to find reward from the tubes located in the center of the display discs, one bee was randomly selected for the training phase, and assigned at random to one of two groups to be trained either to the “larger-than” or “smaller-than” relational rule learning task. During the training phase, an individual bee was trained on six discs (three of one size and three of a different size, but the same type and color) on the back wall of the arena with the same spacing as in pretraining, each displaying one of two differently sized shapes (Figures 1A,C). During each training bout, bees were able to freely land on any stimulus and left the arena when they had fed to satiation. A bout was considered a bee’s visit to the arena, landing on different stimuli until she filled her crop and subsequently returned to her nest. Inter-trial intervals were usually between 5 min and 10 min. Only bees that completed the entire training phase and tests in 1 day were included in the results. As a result, four bees were excluded from the analysis process. During 30 training bouts, one group of bees (*n* = 10) learned that the larger of the two shapes contained 30 μl 50% sucrose solution, and the smaller contained 30 μl saturated quinine hemisulfate solution (larger-than rule). Another group of bees (*n* = 8) learned the reverse contingency (smaller-than rule).

Between training bouts, each disc was rotated pseudo-randomly so that the position of a shape varied across the six discs in relation to the central microcentrifuge tube containing sucrose solution (Figure 1C). The location, shape, and color of stimuli sets were changed between bouts. The shapes used in training varied in size (small, medium, large), type (circle, rectangle, cross), and color (black, green, purple; Figure 1A). Only one type and color of the stimulus was presented to a bee in each bout and only two of the three sizes were presented during one bout. The dimensions of the shapes were as follows: small circle: *Ø* = 1.07 cm; medium circle: *Ø* = 1.97 cm; large circle: *Ø* = 2.87 cm; small rectangle: 0.93 cm × 1.18 cm; medium rectangle: 1.79 cm × 2.92 cm; large rectangle: 2.3 cm ×3.94 cm; small cross: width of bars = 0.46 cm, length of bars = 1.3 cm; medium cross: width of bars = 0.6 cm, length of bars = 2.15 cm; large cross: width of bars = 0.96 cm, length of bars = 2.87 cm. Note that there was large variability between physical features of stimuli (Supplementary Figure S1). For example, the total area of the medium rectangular was larger than the total area of the large cross (see Supplementary Figure S1A). This variability ensured the bees were not able to solve the task by associating an absolute size of stimuli with certain reinforcements. Several stimuli were paired with both positive and negative reinforcements during the training phase. For instance, medium size stimuli were paired with the positive reinforcement in some training bouts while these were paired with negative reinforcement in the rest of the training bouts. All of these variations described ensured that low-level visual cues could not be used to solve the task. Stimuli were cleaned between each training bout with 70% ethanol in water (v/v) to ensure odor cues were not used to solve the task. After the daily experiment, all used microcentrifuge tubes were washed with soap-water, then cleaned with 70% ethanol solution. Finally, they were rinsed with water and air-dried at room temperature during the night.

### Testing Phase

Following the training phase, each bee was tested in the same setup as in training in three different scenarios, but with stimuli in the tests providing 30 μl of sterilized water (Figures 1B,C). Tests lasted 120 s, at which point the bee was gently removed from the arena by using a cup and placed into the corridor until stimuli were changed for the refreshment bouts. Each test was separated by two refreshment training bouts between tests to maintain the bee’s motivation.

The sequence of the three tests was counterbalanced across bees. The learning test evaluated performance by testing bees on one of the same sets of stimuli used during training, pseudo-randomly chosen (i.e., a random number generator was used to generate a random sequence of tests for each bee). The learning test used only the small- and larger-sized training shapes. The other two tests used either a novel shape and size (star) or a novel color (yellow), with the other properties pseudo-randomly chosen. The dimensions of the 5-pointed stars were as follows: small star: length of the side of point = 0.5 cm; large star: length of the side of point = 1.23 cm (see Supplementary Figure S1). As in training, stimuli were cleaned between each bout during the testing phase with 70% ethanol in water (v/v) to ensure odor cues were not used. Trained bees were removed from the nest once the training and test phases were finished.

### Statistical Analysis and Probabilistic Model of the Learning Curve

To evaluate bees’ performance over bouts, the percentage of correct choices (choices were defined as when a bee touched a microcentrifuge tube with her antennae or when she landed on a microcentrifuge tube) was calculated from either all choices or from only the first or second choices within each block of six bouts during training (total of five blocks). Using a generalized linear mixed model (GLMM) for binary probability (correct or incorrect), the effect of different factors such as colony, group of training and interaction between the trial block and group of bees in the bees’ performance were calculated. The bee identity was included in the model as a random factor. GLMMs were performed in MATLAB (MathWorks, Natick, MA, USA).

To determine whether bees used relative size information, rather than any other visual cues, the choices of bees during the unrewarded tests were evaluated by a Wilcoxon signed-rank test. Further, a Kruskal–Wallis test was used to statistically evaluate and compare whether the bees’ performance or choice numbers in different blocks of bouts are from the same distribution.

To test if bees might use a win-stay/lose-switch strategy during training, we calculated the conditional probabilities of each bee’s second choice (*c*_2_) given their first choice (*c*_1_) at each block of 10 bouts. A conditional probability, “Probability of B, given A (*P*{*B*|*A*}),” is a probability of an event (B) occurring given that another event (A) has already occurred. The conditional probability of a lose-switch strategy, i.e., a correct second choice after an incorrect first choice, is calculated by *P*{*c*_2_ = 1|*c*_1_ = 0} = *P*{*c*_2_ = 1, *c*_1_ = 0}/*P*{*c*_1_ = 0} where *P*{*c*_2_ = 1, *c*_1_ = 0} is the joint probability of a correct second choice and an incorrect first choice and *P*{*c*_1_ = 0} is the probability of the first incorrect choice. The conditional probability *P*{*c*_2_ = 1|*c*_1_ = 0} at more than chance level indicates that a bee switched to another presented size when they found the first choice was incorrect. In the same way, we can calculate the conditional probability of a win-stay strategy, using *P*{*c*_2_ = 1|*c*_1_ = 1} = *P*{*c*_2_ = 1, *c*_1_ = 1}/*P*{*c*_1_ = 1}, i.e., the bee’s second choice is the same size as the first choice when their first choice was correct.

### Model of Prediction of Learning Curve Based on a Bee’s First Two Choices

We propose a Markov stochastic model (Gagniuc, 2017) to describe the learning curve of bees’ choices (total choices at each bout) based on the information of two first choices of bees. The performance of the model at each bout is assumed as

$$Perf=P\{c_{1}=p\}\sum_{\text{k}=2}^{N}\overset{\text{k}}{\underset{\text{i}=2}{\Pi }}P\{c_{\text{i+1}}=q|c_{i}=p\}$$

*P*{*c*_1_} is the probability of the first choice at each bout and *P*{c_(i+1)_|*c*_i_} is the conditional probability of (*i* +1) − *th* choices given the of *i* − *th* choices (*i* ≥ 1) for when each choice in the sequential choices is correct or incorrect. *p* or *q* = 1 if the choices are correct, otherwise *p* or *q* = 0. We assume that the conditional probabilities of two sequential choices from the third choices are equal to the conditional probability of the second choice given the first choice expressed by bees at each bout of training. The sequence of possible events in which the probability of each event depends only on the state achieved in the previous event will be stopped (N) when the simulated bees collect all three positive reinforcements along with two, one or no incorrect choices within each bout according to the average number of choices at each bout.

## Results

### Bees’ Overall Performance Increased Over the 30 Training Bouts

**Figure 2:**
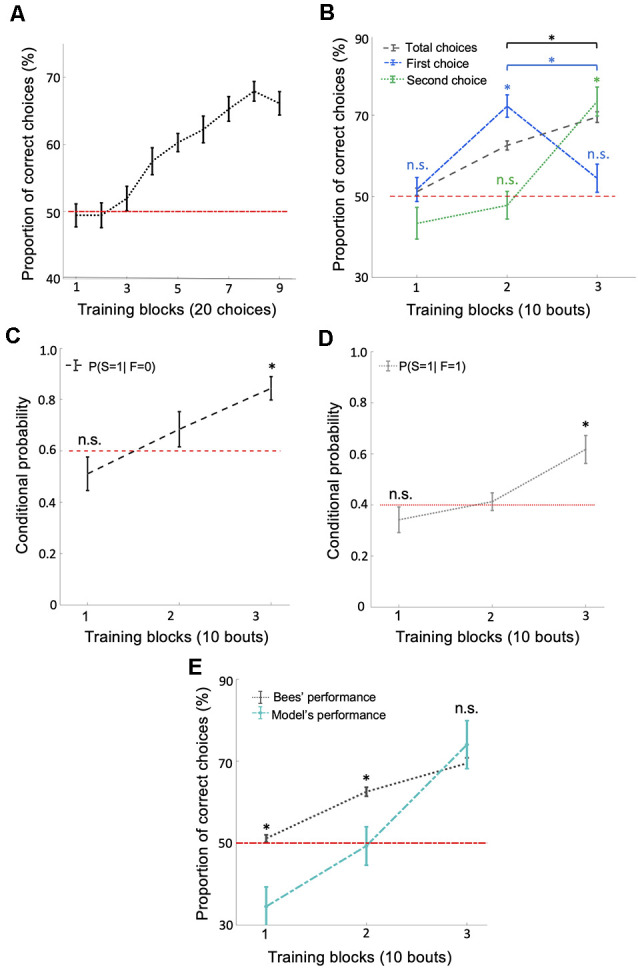
Bees use a win-stay/lose-switch strategy after extensive training. **(A)** There was a significant increase in the number of correct choices over the 180 conditioned choices (*p* = 0.018).** (B)** Bees’ performance over three blocks of 10 training bouts during the relative size discrimination task. Performance increased gradually over bouts when considering the total number of choices in each bout (black dashed line; *p* = 1.96e-4). Bees’ first choice performance increased significantly from the first to the second block of training bouts to 72.22% (*p* = 3.71e-4) but then dropped to chance level from the second to the third block of training bouts (blue dash-dotted line; *p* = 0.79). The second choice performance was near chance for the first two blocks of training bouts (*p* > 0.49) but then increased significantly during the third block of training bouts (green dotted line; *p* = 4.28e-4). These results indicate that bees changed to a win-stay/lose-switch strategy after extensive training. Vertical lines = standard error of the mean. Red dashed line = chance level performance (50%). **(C,D)** The average conditional probabilities of a bee’s second choice within each bout being correct given the outcome of the bee’s first choice of the bout (either correct or incorrect). Both conditional probabilities increased to above chance during the second and third blocks of bouts (*p* = 2.27e-4 for win-stay and *p* = 8.40e-4 for lose-switch). **(E)** Our win-stay/lose-switch model’s performance matches our bees’ performance on the task during the last block of 10 bouts during training (*p* = 0.15), again suggesting that after extensive training bees changed to a win-stay/lose-switch strategy (Vertical lines = standard error of the mean). Red dashed line = chance level. *p < 0.05 and n.s., *p* > 0.05.

A multivariate statistical model, GLMM, applied to the performance of bees demonstrates a significant increase in the proportion of correct choices made over the 180 choices of the training phase (Figure 2A, *p* = 0.018) irrespective of the shape, color or position of patterns within the stimuli. No significant differences were found between the learning curves of the two different contingency groups (i.e., “larger-than” rule vs. “smaller-than” rule; *p* = 0.87). The output of the GLMM confirms that there was no significant difference between the different colonies of bees during the training phase (*p* = 0.37). These results show that bees became better at solving either contingency over training bouts.

### Bees Used a Win-Stay/Lose-Switch Strategy After Extensive Training

The typical analysis used to determine whether an animal has solved a particular task is to calculate the animal’s performance based on the number of correct and incorrect choices throughout the training phase. At first inspection, bees’ behavior during training suggests they learned to solve the concept-based task (Figure 2A). However, a finer examination of their choices suggests the involvement of another strategy in the later stages of training. If bees had only used the concept of relative size throughout training, their first choices should reflect this by increasing in accuracy throughout the 30 bouts. Although bees’ average overall accuracy gradually increased to 70% (significantly above chance level) over the 30 training bouts (Figure 2B; Wilcoxon signed-rank test: *z* = 3.72, *n* = 18, *p* = 1.96e-4), their first-choice accuracy rose to 72% (significantly above chance level: Wilcoxon signed-rank test: *z* = 3.55, *n* = 18, *p* = 3.71e-4) over the first 20 bouts and then decreased to chance level (54%) over the next 10 bouts (Wilcoxon signed-rank test: *n* = 18, *z* = 1.25, *n* = 18, *p* = 0.21; Figure 2B) and decreased significantly across last two blocks of bouts (Wilcoxon signed-rank test: *z* = 2.83, *n* = 18, *p* = 4.59e-3). Second-choice accuracy was not different from chance level during the first two-thirds of the training phase (Wilcoxon signed-rank test: *z* = −0.67, *n* = 18, *p* = 0.49), but increased in the final third of the training phase to 73.33%, significantly above chance level (Figure 2B; Wilcoxon signed-rank test: *z* = 3.52, *n* = 18, *p* = 4.28e-4). These results suggest that bees changed to a win-stay/lose-switch strategy after around 20 bouts of training, i.e., if they find a reward at a stimulus they choose the same type of stimulus next, or if no reward is found at a stimulus they choose a different type of stimulus next (see Supplementary Figure S2 for the individual differences between bees).

To help evaluate the possibility that bees switched strategies partway through training, we calculated the conditional probabilities (see “Materials and Methods” section) for: (1) a correct second choice after a correct first choice (win-stay); and (2) a correct second choice after an incorrect first choice (lose-switch). Both of these two conditional probabilities increased over bouts (Figures 2C,D; Kruskal–Wallis test, chi-sq > 12.94, df = 53, *p* < 1.55e-3), most notably rising to significantly above chance level in the last third of training (Wilcoxon signed-rank test: *z* = 3.68, *n* = 18, *p* = 2.27e-4, chance level = 0.4 for win-stay and *z* = 3.33, *n* = 18, *p* = 8.40e-4, chance level = 0.6 for lose-switch), again suggesting that bees had changed to a win-stay/lose-switch strategy. Note that the chance levels of the conditional probability of correct second choice in a win-stay and lose-switch strategy were 0.4 and 0.6, respectively, because after first choosing correctly, only two of the five remaining stimuli were correct, and after a first incorrect choice, three of the remaining five stimuli were correct.

It may have been that after a first choice, bees simply chose the stimulus nearest to that first choice. To determine whether a bee’s stimulus choice was based on physical closeness to their previous choice, we also evaluated the spatial pattern of their landings. Bees were more likely to choose stimuli further away than those closest to their previous choice (Supplementary Figure S3; Wilcoxon signed-rank test: *z* > 3.72, *n* = 18, *p* < 1.95e-4). Later in training, bees’ second choices were further away from their first choice compared to earlier in training (Supplementary Figure S3; Kruskal–Wallis test, chi-sq = 7.84, *df* = 53, *p* = 0.01). These results indicate that bees did not make their second choice by visiting an adjacent stimulus, but rather searched for specific types of stimuli, following either a relational rule or win-stay/lost-switch strategy.

#### Modeling a Win-Stay/Lose-Switch Strategy

To further examine whether bees switched strategies during training, we utilized a probabilistic model based on a win-stay/lose-switch strategy. Within our model, we used bees’ overall and conditional performance (Figures 2B,E) and initial first and second choices to predict bees’ subsequent choices in each bout (see “Materials and Methods” section). Figure 2E shows that our model predicts the bees’ performance in the last 10 bouts (i.e., no difference between the model’s performance and bee’s performance; Wilcoxon signed-rank test: *z* = −1.41, *n* = 18, *p* = 0.15). In contrast, our model’s predicted performance was significantly poorer than the performance of bees in the first 20 bouts (Wilcoxon signed-rank test: *z* > 2.32, *n* = 18, *p* < 0.01 for both first two blocks). The ability of our model to predict the behavior of our bees in the later stages of training but not the initial stages supports the hypothesis that bees changed to a win-stay/lose-switch strategy within the last 10 bouts of training.

### Bees Retained the Concept of Relative Size After Having Switched Strategies

**Figure 3:**
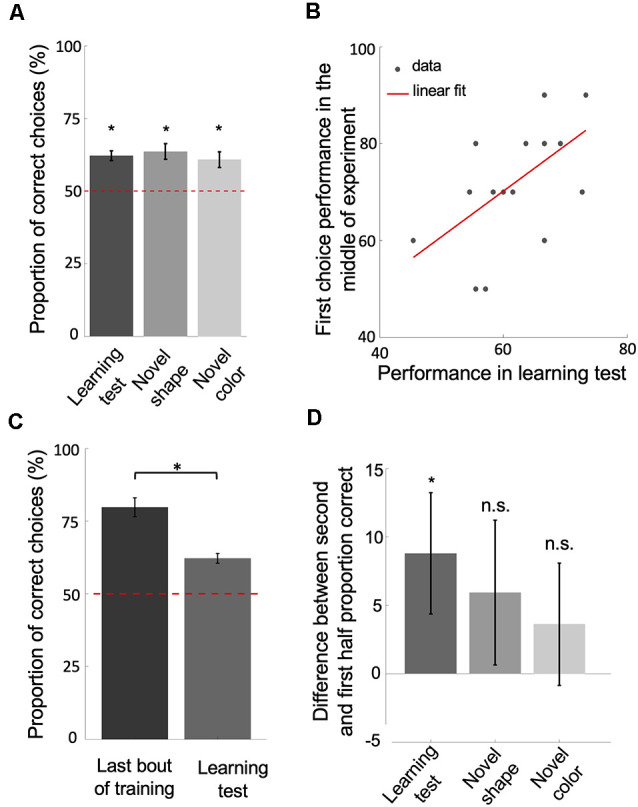
Bees learn and retain a relative size rule. **(A)** The performance of bees during each of the three unrewarded tests shows that they learned and retained the concept of relative size (*p* < 2.41e-3).** (B)** The scatter plot displays the correlation between the performance of bees in the learning test and their first choice performance before changing strategies, during the second block of 10 bouts (rho = 0.58, *p* = 0.01). The red solid line = line of best fit. **(C,D)** The significant drop in performance from the last bout of training to the learning test (*p* = 9.30e-4; **D**) and the difference in performance between the second and first half of choices during each of the tests (*p* = 0.03 for learning test; *p* = 0.28 for novel shape transfer test; *p* = 0.14 for novel color transfer test) suggest that bees had begun the tests with the win-stay/lose-switch strategy. Bars = mean. Vertical lines = standard error of the mean. Red dashed line = chance level (50%). *p < 0.05 and n.s., *p* > 0.05.

So far, our analyses and model results suggest that bees used a win-stay/lose-switch strategy only after extensive training. Bees seemed to have used a different strategy during the initial blocks of training bouts. Their increased performance to above chance level, suggests they were discriminating against the stimuli based on size. To ensure that the bees’ initial strategy had been a relative size rule, we measured bees’ performance directly after training in unrewarded tests. Because the tests were unrewarded, bees could not solve the task based on a win-stay/lose-switch strategy. Bees’ performance on the learning test was above chance level (Wilcoxon signed-rank test: *z* = 3.73, *n* = 18, *p* = 1.87e-4), as was their performance on the novel shape transfer test (Wilcoxon signed-rank test: *z* = 3.51, *n* = 18, *p* = 4.46e-4), and on the novel color transfer test (*z* = 3.03, *n* = 18, *p* = 2.41e-3 for novel color; Figure 3A). Note that the variability in different shape sizes and resulting overlap between sizes across shapes prevented bees from associating a general size with reward (Supplementary Figure S1). These results suggest that the bees had at some point during training learned to solve the task based on the concept of relative size.

Because animals vary in their learning and performance, we posited that if bees had learned and retained a relative size rule, how well they performed in training before changing strategies should reflect how well they perform (i.e., remember the relational rule) during the learning test. In line with this, there was a positive correlation between the average of first choice accuracy in the second third of the training phase (before strategy change) and bees’ performance in the learning test (Figure 3B; Spearman correlation: rho = 0.58, *n* = 18, *p* = 0.01). Although bees seemed to have changed strategies after extensive training, the results of the unrewarded tests show that bees had learned the relative size rule during training, retained the rule even after having changed strategies late in training, and therefore resorted to the relative size rule strategy during the tests.

Note that the performance of bees in the learning test was significantly poorer than the last bout of the training phase (Figure 3C; Wilcoxon signed-rank test: *z* = 3.31, *n* = 18, *p* = 9.30e-4). This suggests that bees began the learning test using a win-stay/lose-switch strategy. This makes sense because they had just been using a win-stay/lost-switch strategy during training and did not know that the test was unrewarded. Further, bees’ performances on the second half of choices during each of the tests was better than their performance on the first half (Figure 3D; Wilcoxon signed-rank test: *z* = 1.82, *n* = 18, *p* = 0.03 for Learning test; *z* = 0.57, *n* = 18, *p* = 0.28 for Novel shape; *z* = 1.05, *n* = 18, *p* = 0.14 for Novel color), indicating that bees had reverted to the retained relative size strategy.

**Figure 4:**
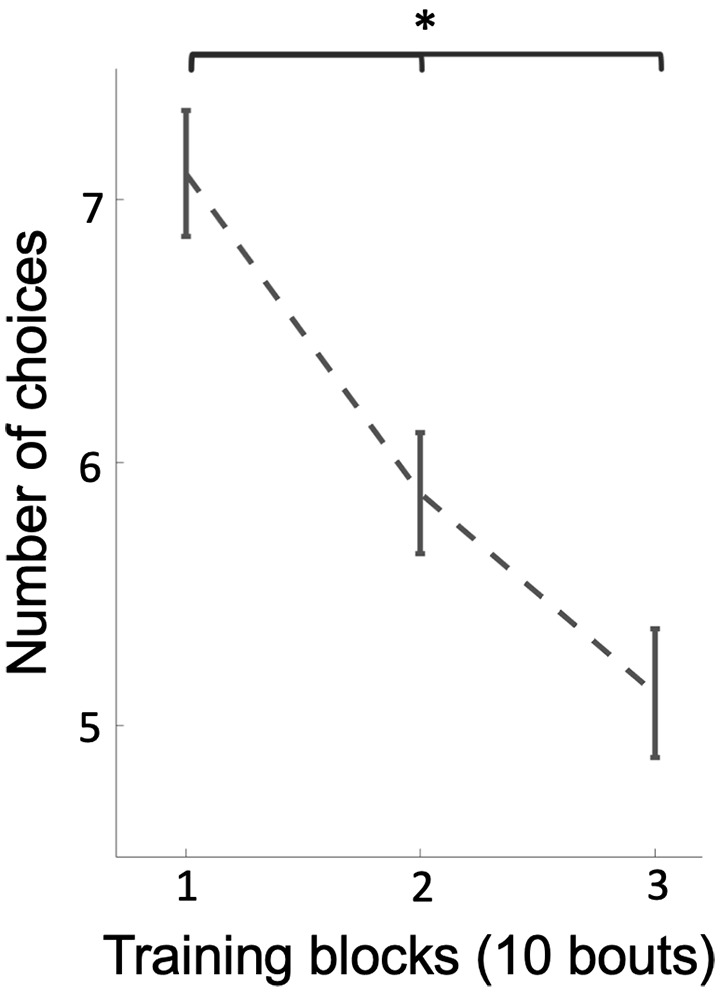
The average of the number of choices on stimuli (correct and incorrect) over three blocks of 10 training bouts. Over training-bouts, bees made fewer choices to visit all three available rewarding stimuli (*p* = 1.17e-5), indicating that bees continually increased efficiency on solving the task during training. Vertical lines = standard error of the mean. *p < 0.05.

Why would bees change strategies if they were already performing above chance level? We hypothesized that bees might change strategies if the new strategy was more efficient, i.e., it took them less effort to locate all three rewarding discs (discs were not refilled during training). In support of this, the number of total choices by bees decreased from an average of 7.1 choices per bout at the beginning of training to an average of 5.1 choices per bout at the end of training (Figure 4; Kruskal–Wallis test, chi-sq = 22.70, *df* = 53, *p* = 1.17e-5), indicating that bees’ efficiency increased during training across a change in strategy.

## Discussion

We demonstrate and corroborate previous findings (Avarguès-Weber et al., 2014; Howard et al., 2017) that bees can learn a relative size rule, but in our study, they opted to use a simpler strategy after extensive training. Because there can often be more than one way of processing the same stimuli to solve a cognitive task, it is useful to examine individual strategies and over extended periods to explore if multiple strategies might be at play. In our paradigm, we prevented bees from using low-level visual cues. Initial increases in performance suggested that bees learned the task and later performance on unrewarded tests verified that bees had learned and retained a relational rule, as was previously demonstrated in honeybees (Avarguès-Weber et al., 2014; Howard et al., 2017). However, statistical analyses showed that after extensive training, bees began to use a win-stay/lose-switch strategy based on whether or not they were rewarded on each stimulus. Bees’ performance calculated by their first choices or by multiple sequential choices revealed a strategy of decision making that had been hidden within the gross calculation by total choices. Averaging all choices in a training bout or test is common within bee cognition and within other animal research communities. We suggest that interpretations of any animal cognition study involving multiple choices include analyses of first and sequential choices to investigate potential alternative strategies.

Theoretical and empirical work maintains that animals tend to follow the “law of least effort” (Hull, 1943; de Froment et al., 2014), whereby subjects choose strategies that minimize the costs in obtaining desirable outcomes (Hull, 1943; Mobbs et al., 2018). In comparative cognition research, animals may use strategies different to those we intend a specific paradigm to test and still perform well on the behavior we are measuring (Shettleworth, 2001; Pfungst, 2010; Chittka et al., 2012; Guiraud et al., 2018; Vasas and Chittka, 2019). Most studies on the “law of least effort” have focused on the idea that animals opt to minimize physical work, but this idea extends to the cognitive effort as well (Elner and Hughes, 1978; Kool et al., 2010; LeDoux, 2012). The ability to change decision-making strategies with the changing demands of the environment is essential to adaptive behavior, and therefore survival. Lloyd and Dayan (2018) proposed that constant monitoring of information to promptly assess and predetermine decision-making strategies would be too costly for animals to maintain. Similarly, commitment for extended periods to one strategy without the ability to adjust could be deleterious (Lloyd and Dayan, 2018). These authors suggested, with support from computational models, that temporal commitment to certain strategies with intermittent interruption to assess costs and switch strategies would be more advantageous for real-world scenarios. Bumblebees in our study seem to follow a similar overall approach, as they first learn an abstract concept (relative size) and stick with this rule for approximately 20 bouts, at which point they change to a new strategy (see Supplementary Figure S2 for the individual difference between bees). A decrease in the number of choices taken to find all rewarding stimuli (Figure 4) indicate that bees may have changed strategies to become more efficient. Further studies are needed to check the role of efficiency in strategy selection in animals. These further studies should involve videotaping the behavior of bees during the training and test phases so that one can make some direct inferences about time invested, mechanisms of inspecting stimuli, and the efficiency of decisions.

In this light, our results support the idea that animals can adaptively weigh the costs of cognitive effort across decision-making approaches and choose the less cognitively demanding strategy (Risko and Gilbert, 2016). This interpretation requires that the win-stay/lose-switch strategy was simpler than the relative size rule. Indeed, the win-stay/lose-switch heuristic is cognitively less demanding than any relational rule, simply because it is based only on the outcome of the previous choice, and therefore could be solved using working memory alone (Nowak and Sigmund, 1993). Accordingly, bees could have stored the visual template of the first stimulus in working memory and, if the first choice was correct, subsequently chosen a stimulus that had more overlap with the stored template, or if the first choice was incorrect, subsequently chosen a stimulus with less overlap (template hypothesis; Dittmar et al., 2010). The win-stay/lose-shift strategy has been broadly observed and explored in bees foraging strategies and flower constancy amongst variable rewarding species of flowers (Greggers and Menzel, 1993; Chittka et al., 1997; Menzel, 2001; Raine and Chittka, 2007; Real, 2012). This type of sequential matching/non-matching to sample strategy is solvable with a simple computational model based on the known neural circuitry of the bee brain, without requiring any higher-order abstract concept (Cope et al., 2018). Learning and applying an abstract concept like relative size requires a substantial abstraction process to different stimuli that must work independently of the physical characteristics of stimuli (Zentall et al., 2008). In mammals, it is assumed that higher cognitive functions processed in the prefrontal cortex or analogous structures are essential for rule learning (Wallis et al., 2001; Miller et al., 2003). In insects, it has been proposed that rule learning occurs in the mushroom bodies, high-level sensory integration centers (Chittka and Niven, 2009; Menzel, 2012). In contrast to rule-learning, bees can use a simple associative mechanism to remember the previously visited stimulus to make decisions about a subsequent stimulus. Therefore, the effort required in a win-stay/lose-switch type mechanism is likely to be lower than an abstract rule because bees can learn to recognize and associate a stimulus with reward without using their mushroom bodies (Devaud et al., 2015; MaBouDi et al., 2017). For example, honeybees with inactivated mushroom bodies can perform some odor learning tasks as well as control bees (Devaud et al., 2015; Carcaud et al., 2016). Further, a realistic computational model of olfactory information processing in the bee brain shows that two parallel odor pathways with different functions provide the flexibility necessary for comparing multiple olfactory stimuli during associative and non-associative discrimination tasks (MaBouDi et al., 2017).

Although our results indicate that bees switched to a win-stay/lose-switch heuristic, it is unclear why bees would learn the relative size concept first if the win-stay/lose-switch strategy is cognitively simpler. We speculate that this strategy may have been initially favored simply to reduce the load on long-term memory and to speed up the decision-making process to avoid the quinine-containing discs. During pretraining, bees only received a reward from white disks. When training began, all of the discs suddenly contained colored shapes and the bees found not only the reward but also aversive quinine. Because of this abrupt and dramatic change, bees’ priority may have been to learn to avoid the quinine containing discs. To accomplish this quickly, they could have extracted a set of elementary visual features to avoid in the first bout of training. During the next bouts, instead of switching to a new strategy relying on working memory, they stuck with identifying and avoiding the template for the quinine containing discs. Over the next trials, they learned to generalize and group visual features across stimuli in a manner consistent with the concept of relative size (Zentall et al., 2008; Avarguès-Weber and Giurfa, 2013). Because constant monitoring of how well they were doing would be too costly (Lloyd and Dayan, 2018), it might have taken them some time to assess their performance and try out a new strategy. Further analysis of bees’ behavior during the training and test phases are required to uncover the true mechanisms underlying bees’ strategy selections.

As a result of bees learning a relative size rule early in training, we would have expected to see an improvement on second choice performance from the first 10 bouts to the second 10 bouts in the training phase similar to the bees’ improvement on first choices (Figure 2B). However, bees’ performance on second choices was not significantly different from a chance level within 20 bouts of training. We are unable to say from our data why this was the case, but speculate that motivation and attention may play a role—once bees found the reward, they might have been less likely to fly back within the arena to view stimuli head on to properly view and assess stimuli, and rather flew directly to a nearby disc to check for food, which statistically would be more likely to be unrewarding (because of the remaining five discs only two would be rewarding). This type of motivational-based exploration may also account for why bees eventually changed to a win-stay/lose-switch strategy. Supplementary Figure S2 shows a large variability between individuals in second choice performance, and therefore individual differences in motivation and attention may have played a part in why second choice performance was lower than expected (Muller et al., 2010; Carere and Locurto, 2011). However, many of the bees did show an improvement in their second choices from the first 10 bouts to the second ten bouts. Analyses of sequential choices in future studies of animal cognition will help resolve these questions.

## Data Availability Statement

The datasets presented in this study can be found in online repositories. The names of the repository/repositories and accession number(s) can be found below: https://figshare.com/s/ebf002a9baa78bc1ef7b.

## Ethics Statement

Ethical review and approval was not required for the animal study because there are currently no international, national, or institutional guidelines for the care and use of bumblebees in research. However, experimental design and procedures were guided by the 3Rs principles (Russell and Burch, 1959). Bumblebees were cared for on a daily basis by trained and competent staff, which included routine monitoring of welfare and provision of correct and adequate food during the experimental period.

## Author Contributions

HM and LC conceived the study. HM designed and performed the experiment. HM and CS analyzed the data. HM, CS, and LC wrote the article.

## Conflict of Interest

The authors declare that the research was conducted in the absence of any commercial or financial relationships that could be construed as a potential conflict of interest.

## References

[B1] Avarguès-Weber A. Giurfa M. (2013). Conceptual learning by miniature brains. Proc. R. Soc. Lond. B Biol. Sci. 280:20131907. 10.1098/rspb.2013.1907 24107530 PMC3813328

[B2] Avarguès-Weber A. d’Amaro D. Metzler M. Dyer A. G. (2014). Conceptualization of relative size by honeybees. Front. Behav. Neurosci. 8:80. 10.3389/fnbeh.2014.00080 24672444 PMC3953954

[B3] Carcaud J. Giurfa M. Sandoz J. C. (2016). Parallel olfactory processing in the honey bee brain: odor learning and generalization under selective lesion of a projection neuron tract. Front. Integr. Neurosci. 9:75. 10.3389/fnint.2015.00075 26834589 PMC4717326

[B4] Carere C. Locurto C. (2011). Interaction between animal personality and animal cognition. Curr. Zool. 57, 491–498. 10.1093/czoolo/57.4.491

[B7] Chittka L. Gumbert A. Kunze J. (1997). Foraging dynamics of bumble bees: correlates of movements within and between plant species. Behav. Ecol. 8, 239–249. 10.1093/beheco/8.3.239

[B6] Chittka L. Niven J. (2009). Are bigger brains better? Curr. Biol. 19, R995–R1008. 10.1016/j.cub.2009.08.023 19922859

[B5] Chittka L. Rossiter S. J. Peter S. Fernando C. (2012). What is comparable in comparative cognition? Philos. Trans. R. Soc. B Biol. Sci. 367, 2677–2685. 10.1098/rstb.2012.0215 22927566 PMC3427551

[B8] Cope A. J. Vasilaki E. Minors D. Sabo C. Marshall J. A. R. Barron A. B. (2018). Abstract concept learning in a simple neural network inspired by the insect brain. PLoS Comput. Biol. 14:e1006435. 10.1371/journal.pcbi.1006435 30222735 PMC6160224

[B12] de Froment A. J. Rubenstein D. I. Levin S. A. (2014). An extra dimension to decision-making in animals: the three-way trade-off between speed, effort per-unit-time and accuracy. PLoS Comput. Biol. 10:e1003937. 10.1371/journal.pcbi.1003937 25522281 PMC4270426

[B9] Devaud J.-M. Papouin T. Carcaud J. Sandoz J.-C. Grünewald B. Giurfa M. (2015). Neural substrate for higher-order learning in an insect: mushroom bodies are necessary for configural discriminations. Proc. Natl. Acad. Sci. U S A 112, E5854–E5862. 10.1073/pnas.1508422112 26460021 PMC4629335

[B10] Dittmar L. Stürzl W. Baird E. Boeddeker N. Egelhaaf M. (2010). Goal seeking in honeybees: matching of optic flow snapshots? J. Exp. Biol. 213, 2913–2923. 10.1242/jeb.043737 20709919

[B11] Elner R. W. Hughes R. N. (1978). Energy maximization in the diet of the shore crab, Carcinus maenas. J. Anim. Ecol. 47, 103–116.

[B13] Gagniuc P. A. (2017). Markov Chains: From Theory to Implementation and Experimentation. Hoboken, NJ: John Wiley and Sons.

[B14] Chittka L. (2017). Bee cognition. Current Biology 27, R1049–R1053. 29017035 10.1016/j.cub.2017.08.008

[B15] Greggers U. Menzel R. (1993). Memory dynamics and foraging strategies of honeybees. Behav. Ecol. Sociobiol. 32, 17–29. 10.1007/bf00172219

[B16] Guiraud M. Roper M. Chittka L. (2018). High-speed videography reveals how honeybees can turn a spatial concept learning task into a simple discrimination task by stereotyped flight movements and sequential inspection of pattern elements. Front. Psychol. 9:1347. 10.3389/fpsyg.2018.01347 30123157 PMC6086205

[B17] Howard S. R. Avarguès-Weber A. Garcia J. Dyer A. G. (2017). Free-flying honeybees extrapolate relational size rules to sort successively visited artificial flowers in a realistic foraging situation. Anim. Cogn. 20, 627–638. 10.1007/s10071-017-1086-6 28374206

[B18] Hull C. L. (1943). Principles of Behavior: An Introduction to Behavior Theory. Oxford, England: Appleton-Century.

[B19] Kool W. McGuire J. T. Rosen Z. B. Botvinick M. M. (2010). Decision making and the avoidance of cognitive demand. J. Exp. Psychol. Gen. 139, 665–682. 10.1037/a0020198 20853993 PMC2970648

[B20] LeDoux J. (2012). Rethinking the emotional brain. Neuron 73, 653–676. 10.1016/j.neuron.2012.02.004 22365542 PMC3625946

[B21] Lloyd K. Dayan P. (2018). Interrupting behaviour: minimizing decision costs *via* temporal commitment and low-level interrupts. PLoS Comput. Biol. 14:e1005916. 10.1371/journal.pcbi.1005916 29338004 PMC5786335

[B220] MaBouDi H. Solvi C. Chittka L. (2020). Bumblebees learn a relational rule but switch to a win-stay/lose-switch heuristic after extensive training. BioRxiv [Preprint]. 10.1101/2020.05.08.085142 32903410 PMC7434978

[B22] MaBouDi H. Shimazaki H. Giurfa M. Chittka L. (2017). Olfactory learning without the mushroom bodies: spiking neural network models of the honeybee lateral antennal lobe tract reveal its capacities in odour memory tasks of varied complexities. PLoS Comput. Biol. 13:e1005551. 10.1371/journal.pcbi.1005551 28640825 PMC5480824

[B23] Menzel R. (2001). “Behavioral and neural mechanisms of learning and memory as determinants of flower constancy,” in Cognitive Ecology of Pollination, eds Chittka L. Thomson J. D. (New York, NY: Cambridge University Press), 21–40.

[B24] Menzel R. (2012). The honeybee as a model for understanding the basis of cognition. Nat. Rev. Neurosci. 13, 758–768. 10.1038/nrn3357 23080415

[B25] Miller E. K. Nieder A. Freedman D. J. Wallis J. D. (2003). Neural correlates of categories and concepts. Curr. Opin. Neurobiol. 13, 198–203. 10.1016/s0959-4388(03)00037-0 12744974

[B26] Mobbs D. Trimmer P. C. Blumstein D. T. Dayan P. (2018). Foraging for foundations in decision neuroscience: insights from ethology. Nat. Rev. Neurosci. 19:419. 10.1038/s41583-018-0010-7 29752468 PMC6786488

[B27] Muller H. Grossmann H. Chittka L. (2010). ‘Personality’ in bumblebees: individual consistency in responses to novel colours? Anim. Behav. 80, 1065–1074. 10.1016/j.anbehav.2010.09.016

[B28] Nowak M. Sigmund K. (1993). A strategy of win-stay, lose-shift that outperforms tit-for-tat in the prisoner’s dilemma game. Nature 364, 56–58. 10.1038/364056a0 8316296

[B29] Perry C. J. Barron A. B. (2013). Honey bees selectively avoid difficult choices. Proc. Natl. Acad. Sci. U S A 110, 19155–19159. 10.1073/pnas.1314571110 24191024 PMC3839751

[B30] Pfungst O. (2010). Clever Hans (The Horse of Mr. Von Osten) A contribution to Experimental Animal and Human Psychology. Available online at: http://www.gutenberg.org/ebooks/33936. Accessed December 1, 2018.

[B31] Raine N. E. Chittka L. (2007). Flower constancy and memory dynamics in bumblebees (Hymenoptera: Apidae: Bombus). Entomol. Gen. 29, 179–199. 10.1127/entom.gen/29/2007/179

[B32] Real L. (2012). Pollination Biology. Elsevier: Academic Press.

[B33] Risko E. F. Gilbert S. J. (2016). Cognitive offloading. Trends Cogn. Sci. 20, 676–688. 10.1016/j.tics.2016.07.002 27542527

[B320] Russell W. M. S. Burch (1959). The Principles of Humane Experimental Technique. London: Methuen.

[B34] Shettleworth S. J. (2001). Animal cognition and animal behaviour. Anim. Behav. 61, 277–286. 10.1006/anbe.2000.1606

[B35] Skorupski P. MaBouDi H. Dona H. S. G. Chittka L. (2018). Counting insects. Philos. Trans. R. Soc. Lond. B Biol. Sci. 373:20160513. 10.1098/rstb.2016.0513 29292360 PMC5784040

[B36] Stach S. Giurfa M. (2005). The influence of training length on generalization of visual feature assemblies in honeybees. Behav. Brain Res. 161, 8–17. 10.1016/j.bbr.2005.02.008 15904705

[B37] Vasas V. Chittka L. (2019). Insect-inspired sequential inspection strategy enables an artificial network of four neurons to estimate numerosity. iScience 11, 85–92. 10.1016/j.isci.2018.12.009 30590253 PMC6308245

[B38] Wallis J. D. Anderson K. C. Miller E. K. (2001). Single neurons in prefrontal cortex encode abstract rules. Nature 411, 953–956. 10.1038/35082081 11418860

[B39] Wasserman E. A. Zentall T. R. (2006). Comparative Cognition: Experimental Explorations of Animal Intelligence. New York, NY: Oxford University Press.

[B40] Zentall T. R. Wasserman E. A. Lazareva O. F. Thompson R. K. R. Rattermann M. J. (2008). Concept learning in animals. Comp. Cogn. Behav. Rev. 3:2 10.3819/ccbr.2008.30002

