# Supplementary material for: Bumblebees Learn a Relational Rule but Switch to a Win-Stay/Lose-Switch Heuristic After Extensive Training
Source: Front Behav Neurosci. 2020 Aug 12;14:137. doi: 10.3389/fnbeh.2020.00137 (PMC7434978; doi:10.3389/fnbeh.2020.00137)
Supplement: Supplementary file 1 [file Table_1.pdf]

Supplementary Material

Supplementary Figures and Tables

(A)

	Total area (cm^2)	Edge length (cm)	Convex hull (cm^2)
Small circle	0.89	3.36	0.89
Medium circle	3.04	6.18	3.04
Large circle	6.46	9.01	6.46
Small rectangle	1.09	4.22	1.09
Medium rectangle	5.22	9.42	5.22
Large rectangle	9.06	12.48	9.06
Small cross	0.98	5.20	2.54
Medium cross	2.22	8.60	3.15
Large cross	4.50	11.44	6.42
Small star	0.56	5.00	1.10
Large star	3.45	12.30	6.74

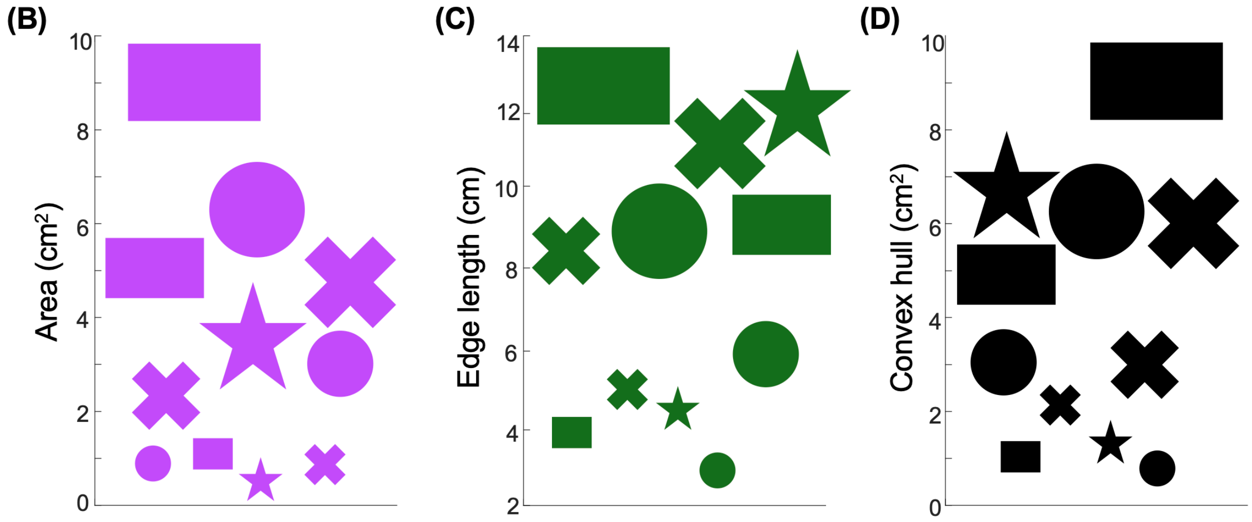

Supplementary Figure S1. Physical features of stimuli (**A**) Calculated total area, edge length and convex hull of each stimulus used in the study. (**B-D**) Plots visualizing variations of each stimulus feature across shapes. Note that for each physical feature, several certain sized stimuli were closer in size to a different size of another shape. For example, the large star was closer in total area to the medium-sized circle and cross, while the medium-sized rectangle was similar to the large circle and cross. This variation prevented bees from associating sizes with reward and required the bees to compare relative sizes amongst shapes presented during each bout.

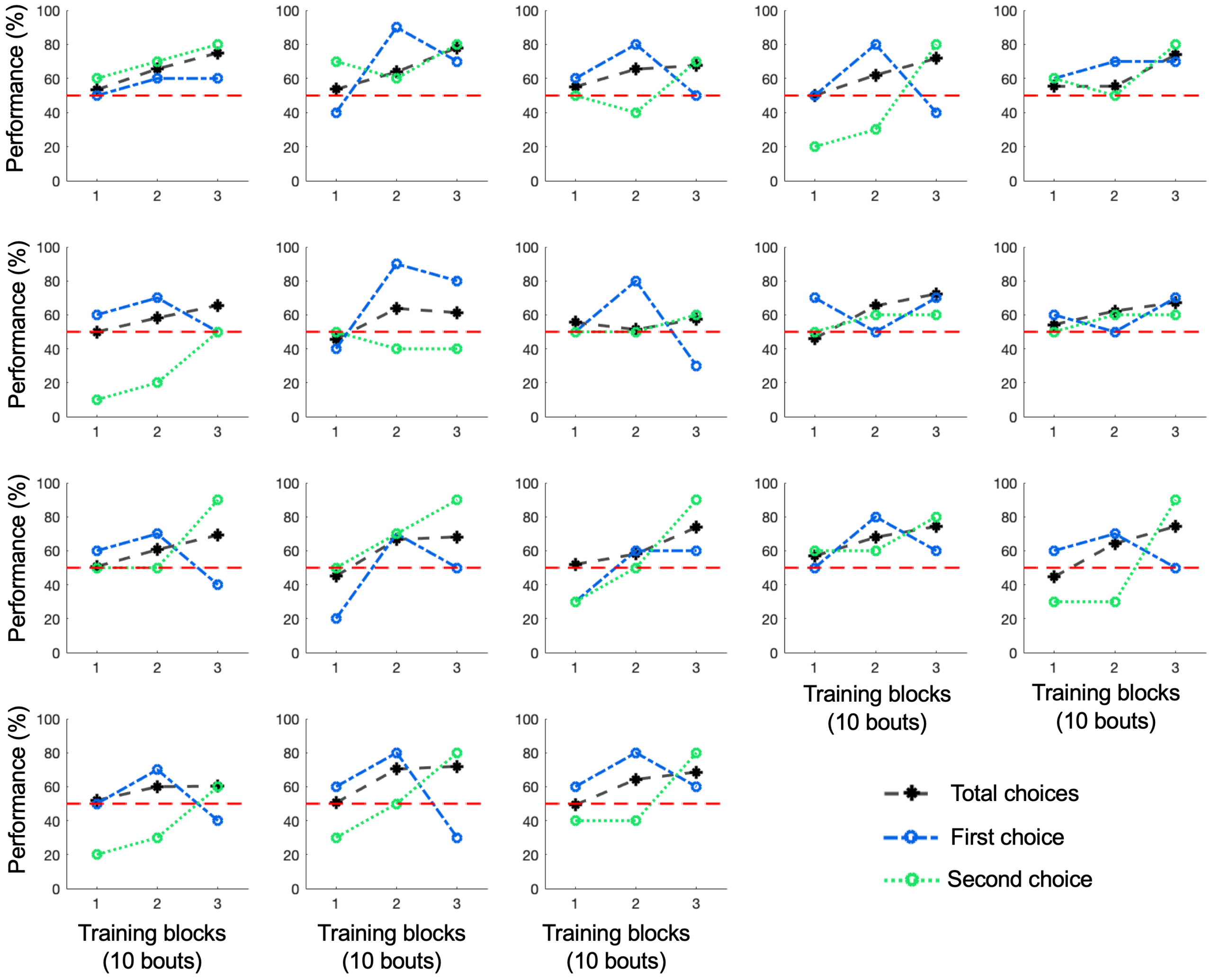

Supplementary Figure S2. Performances of individual bees in the training phase plotted for three different conditions. Black dashed lines show the learning curve of each bee based on the total choices. The blue dash-dotted and green dotted lines display the bees' performances calculated from the first and second choices within each bout, respectively. Red dashed line = chance level (50%).

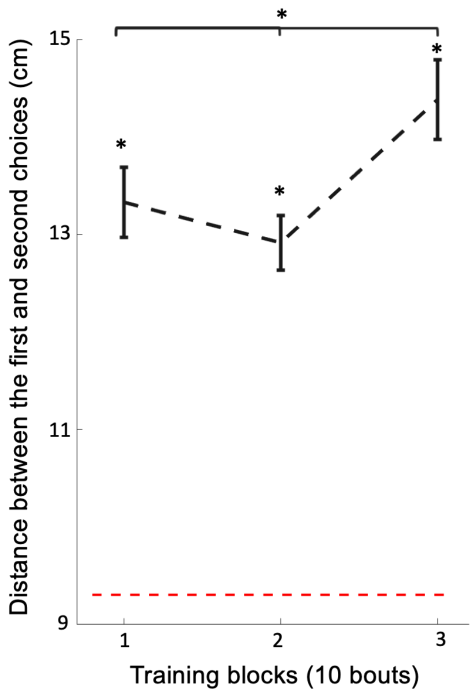

Supplementary Figure S3. Distance between the first and second stimulus choices. Across training, bees were more likely to choose stimuli further away from their first choice than those closer (Wilcoxon signed rank test: $z > 3.72$, $n = 18$, $p < 1.95e\text{-}4$). Over training bouts, the distance between bees' first and second stimulus choices increased (Kruskal-Wallis test, chi-sq = 7.84, df = 53, p = 0.01). The red dashed line indicates the minimum distance between stimuli. Vertical lines = standard error of the mean. * indicates p<0.05.
